# Physical activity intensity and cardiovascular disease in Southwest China: a study on data from the China Multi-Ethnic Cohort study

**DOI:** 10.3389/fcvm.2026.1834010

**Published:** 2026-06-30

**Authors:** Yanjiao Wang, Likun He, Yanjiao Luo, Ying Qian, Jizhuo Yang, Xuehui Zhang, Jianzhong Yin

**Affiliations:** 1 Key Laboratory of Nutrition and Food Safety of Yunnan Provincial Education Department, School of Public Health, Kunming Medical University, Kunming, China; 2 Yunnan Provincial Key Laboratory of Public Health and Biosafety, School of Public Health, Kunming Medical University, Kunming, China; 3 Lincang City Center for Disease Control and Prevention, Lincang, China; 4 Healthy Yunnan Development Think Tank, Kunming, China

**Keywords:** cardiovascular disease, metabolic equivalent, physical activity, plateau environment, the China Multi-Ethnic Cohort

## Abstract

**Objective:**

This study aimed to assess the relationship between physical activity intensity and cardiovascular disease (CVD) in Yunnan Province.

**Methods:**

This cross-sectional analysis was conducted using data extracted from the China Multi-Ethnic Cohort (Yunnan region) study and included 10,477 participants aged 30–79 years. Physical activity intensity was categorized as low (LPA), moderate (MPA), or vigorous (VPA). The primary outcome was composite prevalent CVD, including coronary heart disease, stroke, rheumatic heart disease, pulmonary-derived heart disease, and high-altitude heart disease, which were identified based on self-reported questionnaire data. Participants were diagnosed with CVD if they had one or more of these conditions.

**Results:**

Among the 10,447 participants, 267 (2.56%) had CVD. In the crude model, VPA was associated with lower odds of CVD than LPA [OR (95% CI): 0.10 (0.04, 0.26); *P*-trend < 0.001]. Compared to LPA, VPA was associated with lower odds of CVD [OR (95% CI): 0.30 (0.11, 0.85), *P*-trend = 0.018] after adjusting for age and sex. A similar association was observed after further adjustment for body mass index, waist circumference, smoking, alcohol consumption, education status, diabetes mellitus, hypertension, triglycerides, total cholesterol, high-density lipoprotein cholesterol, low-density lipoprotein cholesterol, total energy intake, total vegetable and fruit intake, and vegetable oil intake [OR (95% CI): 0.27 (0.09, 0.80); *P*-trend = 0.025]. We also conducted stratified analyses according to potential risk factors. We found a significant interaction between waist circumference and the intensity of physical activity regarding the odds of CVD.

**Conclusions:**

VPA was cross-sectionally associated with lower odds of prevalent CVD in this high-altitude Chinese population. These findings should be interpreted with caution due to the cross-sectional design and potential reverse causation.

## 1 Introduction

Cardiovascular disease (CVD) remains the leading cause of morbidity and mortality worldwide, and its burden has been rising rapidly in China, particularly in the less-developed southwestern regions (1, 2). Physical activity (PA) is a well-established modifiable protective factor against CVD, with a documented inverse dose-response relationship (3). However, the majority of previous studies have focused on total PA volume or moderate-to-vigorous PA, whereas the independent role of distinct intensity levels (light, moderate, and vigorous) has been less systematically examined in high-altitude populations.

Chronic exposure to high altitudes induces a series of physiological adaptations that may increase cardiovascular vulnerability. Hypoxia stimulates erythropoiesis, which leads to erythrocytosis, increased blood viscosity, and elevated pulmonary artery pressure (4–6). These mechanisms have been linked to a higher risk of pulmonary hypertension, heart failure, and thromboembolic events in long-term high-altitude residents (7). Therefore, identifying modifiable protective factors such as PA intensity is particularly relevant for this population. A representative highland setting is the Lijiang area in Yunnan Province (average altitude of approximately 2,200 m), where daily activities are shaped by the mountainous terrain and often include walking on slopes, climbing stairs, and agricultural labor. Residents typically engage in a wide range of PA intensities, such as slow walking, household chores, brisk walking, farming, carrying light loads, heavy manual labor, uphill carrying, and strenuous exercise.

The relationship between PA intensity and CVD may be modified by altitude-related physiological adaptations, occupational demands, and lifestyle factors that differ substantially among lowland populations (7). Previous studies have demonstrated a notable clustering of CVD risk factors, such as hypertension and physical inactivity, in Yunnan Province (8, 9), further highlighting the necessity for intensity-specific PA investigations tailored to highland populations. However, few large-scale studies have quantified the association between moderate and vigorous PA against a light intensity reference in such settings.

To address these gaps, this study analyzed data from the China Multi-Ethnic Cohort (CMEC) study and focused on participants from the Lijiang region. We classified PA into the following three levels: low PA (LPA), moderate PA (MPA), and vigorous PA (VPA). We aimed to investigate the associations of MPA and VPA with CVD.

## 2 Materials and methods

### 2.1 Study population

We used data from the CMEC study. The CMEC study was a population-based study conducted from May 2018 to September 2019 that aimed to address the urgent need to understand the risk factors for chronic non-communicable diseases in Southwest China. The rationale, study design, and methods have been previously described (10). Briefly, the cohort was established in five provinces in Southwest China. A stratified multistage cluster sampling method was used to recruit the participants. One or two settlements were selected for each ethnic group, and one to eight communities were selected by the local center for Disease Control and Prevention for every settlement. During sampling, settlements in plateaus, rural areas, basins, and air-polluted regions were considered to represent the variation. The data used in this current study were derived from a baseline CMEC survey. For CMEC (Yunnan region), a survey was conducted in Lijiang City (average altitude of approximately 2,200 m) using a standardized approach and stringent quality control measurements. The survey also included a face-to-face electronic questionnaire, anthropometric measurements, and medical history (11). Trained investigators collected all the data. The inclusion criteria for the current study were as follows: (1) permanent residents who could complete the survey, (2) completed questionnaire interviews, (3) participants who had PA-related data, and (4) no missing data regarding the determination of CVD status. The exclusion criterion was missing PA or outcome data (*n* = 23). Finally, 10,447 participants were included in the analysis. All the participants provided written informed consent. This study was approved by the Medical Ethics Review Board of Kunming University.

### 2.2 Exposure assessment

The information on PA for each participant was collected through the questionnaire during the survey. Both the PA intensity and the amount of PA 1 year preceding the survey were obtained. The PA intensity was represented by the corresponding metabolic equivalent values (MET). This study assigned different MET to different physical activities (Supplementary Table S1). PA was calculated in four domains, namely, leisure, work, transportation, and housework. The sum of activity in each domain was the total PA for each participant. Based on the 2024 Compendium of Physical Activities (12), PA intensity was categorized as LPA (MET < 3), MPA (MET 3–6), or VPA (MET ≥ 6).

### 2.3 Covariate assessment

Data on demographics, lifestyle, and medications were obtained from questionnaires. Trained investigators measured anthropometric indices. A food frequency questionnaire (FFQ) was used to assess habitual diets and determine the consumption of each food group. The reproducibility and validity of the FFQ were evaluated by repeated FFQs and 24-h dietary recalls. Smoking was defined as smoking at least 100 cigarettes. Alcohol consumption was divided into two categories: non-drinkers and drinkers (occasional, seasonal, monthly, or weekly). Education was categorized as high school or below (including no formal education, primary school, junior high school, and high school) or beyond high school (including upper high school, or college and above). Body mass index (BMI) was calculated as body weight in kilograms divided by the square of height in meters. Waist circumference (WC) was measured twice to the nearest 0.1 cm with clothes removed, and the average values were taken. Blood samples were collected after overnight fasting. Fasting serum glucose levels were evaluated using a hexokinase assay. Total cholesterol (TC), low-density lipoprotein cholesterol (LDL-C), and high-density lipoprotein cholesterol (HDL-C) levels were determined using enzyme colorimetry (Beckman). The triglyceride (TG) levels were measured using a glycerol phosphate oxidase-peroxidase reagent (Beckman). Diabetes was defined as a fasting blood glucose (FBG) ≥ 7.0 mmol/L or a 2-h oral glucose tolerance test concentration ≥ 11.1 mmol/L and/or taking glucose-lowering medication(s) (13). Blood pressure was measured three times, and the mean values were used for the analysis. Hypertension was defined as systolic blood pressure ≥ 140 mm Hg and/or diastolic blood pressure ≥ 90 mm Hg or taking antihypertensive drugs (14).

### 2.4 Outcome definition

The primary outcome was composite prevalent CVD, including coronary heart disease, stroke, rheumatic heart disease, pulmonary-derived heart disease, or high-altitude heart disease, which were identified based on a self-reported questionnaire asking whether the participants had ever been diagnosed with coronary heart disease, stroke, rheumatic heart disease, pulmonary-derived heart disease, or high-altitude heart disease by a doctor from second-level and above hospitals. Participants were diagnosed with CVD if they had one or more of these conditions. The validity of self-reported data for CVD has been verified with an accuracy of approximately 97%, and this approach has been used in many population-based studies (15).

### 2.5 Statistical analysis

The normality of continuous variables was determined using the Kolmogorov–Smirnov test. The participants’ characteristics were compared using the *t*-test and *χ*^2^ test for continuous and categorical variables, respectively. Cardiometabolic risk factors were examined across PA intensity levels using a general linear model. Binary logistic regression was used to analyze the relationship between PA intensity and CVD. Several models were examined in this study. Model 1 was a crude model. Model 2 included age and sex. Model 3 included age, sex, BMI, WC, smoking, alcohol consumption, education status, diabetes mellitus, hypertension, TG, TC, HDL-C, LDL-C, total energy intake, total vegetable and fruit intake, and vegetable oil intake. Regression diagnostics were performed to test for collinearity. No significant collinearity was observed among the variables included in the models.

Previous studies have shown that the prevalence of CVD exhibits a sex difference, with men having a higher prevalence of CVD than women (16). Therefore, we conducted a sex-stratified analysis. We also performed analyses stratified by following factors: age (<60 or ≥60 years), BMI (<24 kg/m^2^, ≥24 kg/m^2^), WC (male < 90 cm and female < 80 cm or male ≥ 90 cm and female ≥ 80 cm), smoking status (current and never or ever), alcohol consumption (never, sometimes or always), hypertension (yes or no), and diabetes mellitus (yes or no). Multiplicative interactions were assessed by adding interaction terms to the models to evaluate the interactions between total PA and these factors. Restricted cubit spline (RCS) was used to model the non-linear association between total physical activity and CVD. Statistical analyses were performed using SPSS 25.0 for Windows (IBM Corporation, USA). All statistical tests were two-sided, and we considered *P* < 0.05 to be statistically significant.

## 3 Results

### 3.1 Descriptive characteristics

Of the 10,447 participants (3,650 men and 6,797 women), 267 (2.56%) had CVD. The characteristics of the participants with and without CVD are shown in Table 1. Participants with CVD were more likely to be older and obese. Moreover, they were more likely to have central obesity; a low level of education; more unfavorable lipid profiles, including higher TG and TC and lower HDL-C levels; and low levels of total PA. Regarding dietary factors, participants with CVD tended to consume fewer fruits and vegetables and have a lower total energy intake. However, they consumed more vegetable oil. Total PA was significantly associated with WC and systolic blood pressure (Table 2).

**Table 1:** General characteristics of participants with and without CVD from the China Multi-Ethnic Cohort, Yunnan region.

Characteristic	Without CVD (*n* = 10,180)	With CVD (*n* = 267)	*P*-value
Demographic and lifestyle variables			
Age (years)	52.21 ± 10.32	61.80 ± 9.01	<0.001
Male	3,589 (35.26)	106 (39.70)	0.122
BMI (kg/m^2^)	22.67 ± 3.12	23.29 ± 3.31	0.007
Waist circumference (cm)	77.19 ± 9.28	80.75 ± 10.31	<0.001
Current smoker	2,332 (22.91)	55 (20.59)	<0.001
Drinking	1,390 (13.65)	26 (9.74)	0.183
Education beyond high school	1,035 (10.17)	19 (7.12)	0.033
Disease status			
Diabetes mellitus	439 (4.31)	30 (11.23)	<0.001
Hypertension	2,891 (28.40)	161 (60.30)	<0.001
Glucose and lipid profiles			
FBG (mmol/L)	4.94 (4.62, 5.32)	4.97 (4.64, 5.41)	0.211
TG (mmol/L)	1.50 (1.08, 2.23)	1.68 (1.22, 2.51)	0.002
TC (mmol/L)	4.92 (4.35, 5.58)	5.11 (4.88, 5.90)	0.004
HDL-C (mmol/L)	1.46 (1.21, 1.73)	1.40 (1.13, 1.68)	0.048
LDL-C (mmol/L)	2.93 (2.42, 3.48)	3.04 (2.33, 3.70)	0.413
Dietary factors			
Total energy intake (kcal/day)	1,912 (1,536, 2,350)	1,708 (1,329, 2,109)	<0.001
Total vegetable and fruit intake (g/day)	400 (271, 507)	350 (200, 500)	0.006
Vegetable oil intake(g/day)	49 (32, 68)	51 (32, 82)	0.041
Red meat intake (g/day)	50 (28, 100)	50 (28, 100)	0.298
Total physical activity (METs/day)			
LPA	1.80 (1.43, 2.57)	1.80 (1.31, 2.50)	<0.001
MPA	4.60 (3.80, 5.40)	4.60 (3.90, 5.04)	<0.001
VPA	39.80 (25.75, 53.05)	26.64 (15.33, 41.12)	<0.001

Normally distributed continuous variables are shown as mean ± SD and were compared using the *t*-test. Non-normally distributed continuous variables are shown as median (interquartile range: P25, P75). Categorical variables are reported as counts and percentages [*n* (%)] and were compared using the *χ*^2^ test. CVD, cardiovascular disease; BMI, body mass index; FBG, fasting blood glucose; TG, triglycerides; LDL-C, low-density lipoprotein cholesterol; TC, total cholesterol; HDL-C, high-density lipoprotein cholesterol; METs, metabolic equivalent of task; LPA, low physical activity; MPA, moderate physical activity; VPA, vigorous physical activity.

**Table 2:** Cardiometabolic risk factors of the participants according to total physical activity intensity level.

Risk factor	Total physical activity intensity level			*P* for trend
	LPA	MPA	VPA	
Adiposity				
BMI (kg/m^2^)	22.87 ± 4.21	22.60 ± 3.38	22.69 ± 3.12	0.930
WC (cm)	79.42 ± 10.27	81.41 ± 10.24	77.24 ± 9.31	<0.001
Lipid profiles				
TG (mmol/L)	1.71 (1.13, 2.14)	1.44 (1.14, 2.17)	1.51 (1.08, 2.23)	0.866
TC (mmol/L)	4.77 (4.29, 5.90)	4.91 (4.44, 5.53)	4.93 (4.35, 5.59)	0.884
HDL-C (mmol/L)	1.34 (1.20, 1.67)	1.40 (1.13, 1.74)	1.46 (1.21, 1.73)	0.397
LDL-C (mmol/L)	2.98 (2.48, 3.67)	2.86 (2.45, 3.71)	2.93 (2.41, 3.48)	0.709
Glucose homeostasis				
FBG (mmol/L)	5.31 ± 2.21	5.44 ± 2.12	5.15 ± 1.23	0.072
Blood pressure				
Systolic (mmHg)	134.83 ± 23.46	126.62 ± 23.53	121.96 ± 18.64	<0.001
Diastolic (mmHg)	81.94 ± 13.02	78.80 ± 13.64	79.32 ± 11.35	0.508

Values are shown as mean ± SD. LPA, low physical activity; MPA, moderate physical activity; VPA, vigorous physical activity; BMI, body mass index; WC, waist circumference; TG, triglycerides; TC, total cholesterol; LDL-C, low-density lipoprotein cholesterol; HDL-C, high-density lipoprotein cholesterol; FBG, fasting blood glucose.

### 3.2 Association between PA intensity and CVD

The ORs (and 95% CIs) for CVD according to PA intensity are shown in Table 3. In the crude model, VPA was associated with lower odds of CVD than LPA [0.10 (0.04, 0.26); *P*-trend < 0.001]. Compared with LPA, VPA was associated with lower odds of CVD [0.30 (0.11, 0.85), *P*-trend = 0.018] after adjusting for age and sex. A similar association was observed in the multivariate adjusted model. The adjusted OR (95% CI) was 0.27 (0.09, 0.80) for VPA (*P*-trend = 0.025) after additional adjustment for BMI, WC, smoking, alcohol consumption, education status, diabetes mellitus, hypertension, TG, TC, HDL-C, LDL-C, total energy intake, total vegetable and fruit intake, and vegetable oil intake.

**Table 3:** Adjusted odds ratios (95% CI) for CVD according to total physical activity level.

Model	ORs (95% CI) for odds of CVD			*P* for trend
	LPA	MPA	VPA	
Model 1	1 (ref.)	0.44 (0.13, 1.43)	0.10 (0.04, 0.26)	<0.001
Model 2	1 (ref.)	0.56 (0.17, 1.91)	0.30 (0.11, 0.85)	0.018
Model 3	1 (ref.)	0.46 (0.13, 1.66)	0.27 (0.09, 0.80)	0.025

Model 1: crude ratio.

Model 2: adjusted for age and sex.

Model 3: adjusted for age, sex, body mass index, waist circumference, smoking, alcohol consumption, education status, diabetes mellitus, hypertension, triglycerides, total cholesterol, high-density lipoprotein cholesterol, low-density lipoprotein cholesterol, total energy intake, total vegetable and fruit intake, and vegetable oil intake. OR, odds ratio; ref., reference; LPA, low physical activity; MPA, moderate physical activity; VPA, vigorous physical activity.

**Figure 1:**
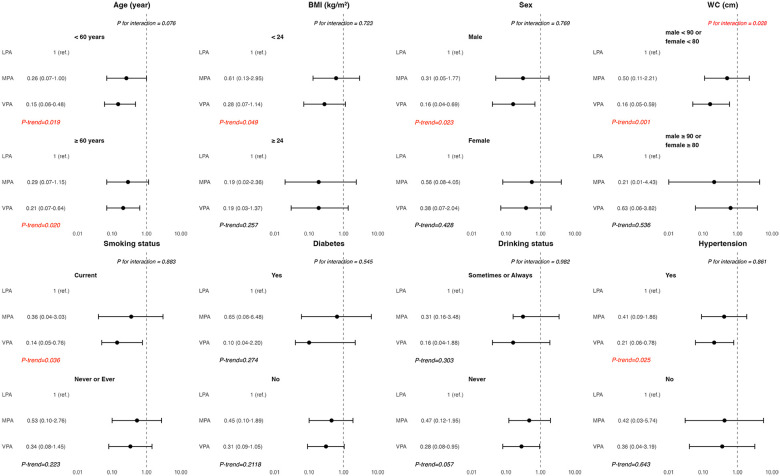
Stratified analyses of the odds of CVD according to physical activity intensity. Results were adjusted for age (if appropriate), sex (if appropriate), body mass index (if appropriate), waist circumference (if appropriate), smoking (if appropriate), drinking (if appropriate), education status, diabetes mellitus (if appropriate), hypertension (if appropriate), triglycerides, total cholesterol, high-density lipoprotein cholesterol, low-density lipoprotein cholesterol, total energy intake, total vegetable and fruit intake, and vegetable oil intake.

**Figure 2:**
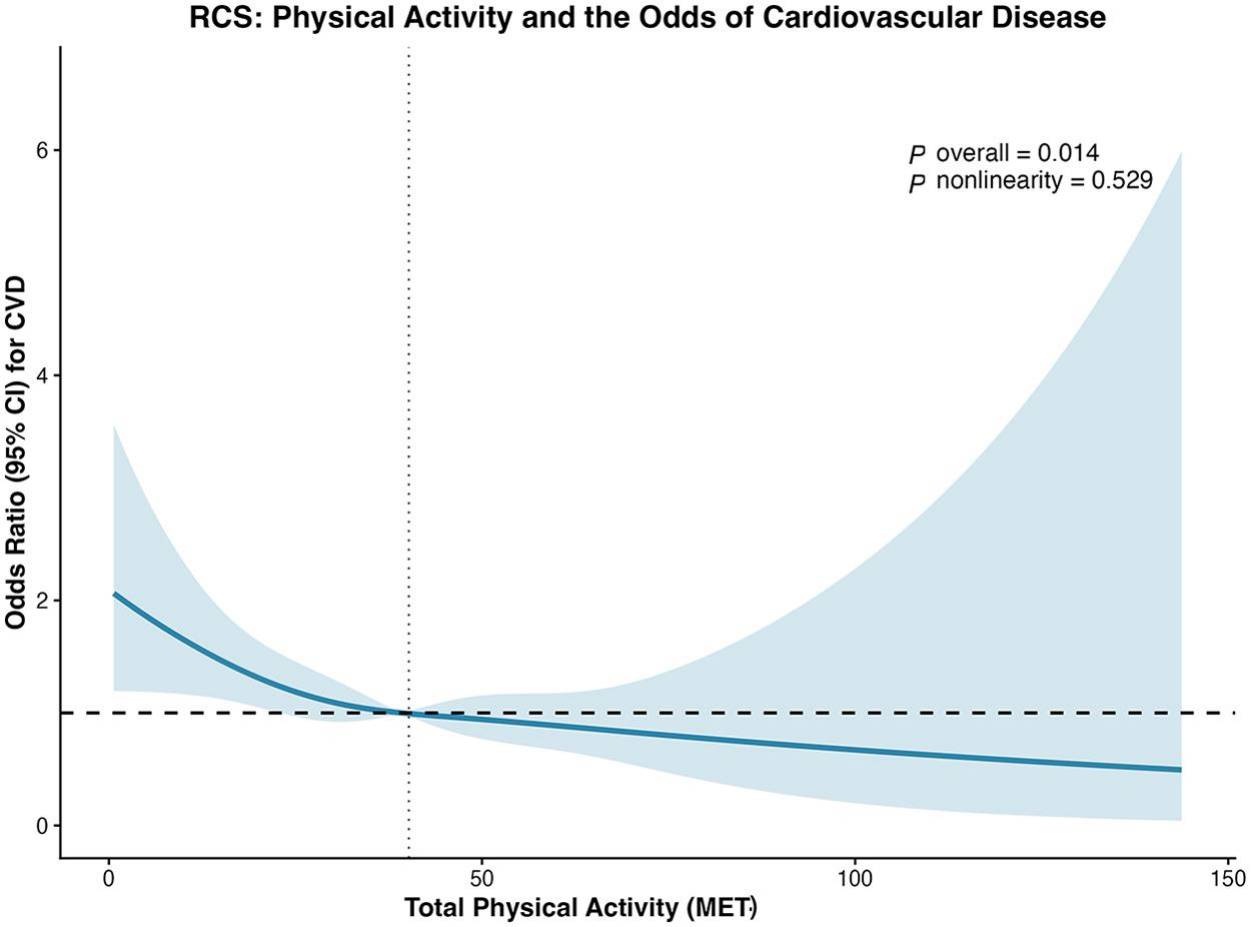
Non-linear relationship between total physical activity and CVD among all participants. In the RCS, the model was adjusted for age, sex, body mass index, waist circumference, smoking, alcohol consumption, education status, diabetes mellitus, hypertension, triglycerides, total cholesterol, high-density lipoprotein cholesterol, low-density lipoprotein cholesterol, total energy intake, total vegetable and fruit intake, and vegetable oil intake. The odds ratios are represented by the solid blue line and 95% CIs by the shaded area. The black horizontal dashed lines represent the reference line y = 1.

We also conducted stratified analyses of potential risk factors, including age, sex, BMI, WC, smoking status, alcohol consumption, diabetes mellitus, and hypertension (Figure 1). We found a significant interaction between WC and PA intensity regarding the odds of CVD. When stratified by central obesity status, the association between PA intensity and CVD was markedly different across WC subgroups. In participants with normal WC (male < 90 cm and female < 80 cm), there was a clear inverse trend between increasing PA intensity and CVD (*P*-trend = 0.001), with VPA showing a substantially lower adjusted OR of 0.16 (95% CI: 0.05, 0.59) relative to LPA. In contrast, among those with increased WC (male ≥ 90 cm and female ≥ 80 cm), the trend was no longer statistically significant (*P*-trend = 0.536), and the OR for VPA vs. LPA was 0.63 (95% CI: 0.06, 3.82). Using RCS models, we examined the possible non-linear relationship between total physical activity and CVD in all the subjects and found no significant non-linear relationship (Figure 2; *P* for non-linearity = 0.529).

## 4 Discussion

In this cross-sectional study of 10,447 adults residing in the high-altitude region of Lijiang, Yunnan Province (average altitude of 2,200 m), we observed that higher total PA levels were associated with lower odds of developing CVD. Notably, compared with LPA, VPA but not MPA, was related to lower odds of CVD after comprehensive adjustment for demographic, lifestyle, clinical, and dietary covariates. Furthermore, a significant interaction was observed between PA intensity and WC. These findings extend the current evidence by demonstrating that VPA was cross-sectionally associated with lower prevalent CVD (17, 18).

Although the observed CVD prevalence in our sample (2.56%) was not exceptionally high compared to national figures, the physiological vulnerability of plateau residents to hypoxia-related cardiovascular stress underscores the public health importance of promoting VPA in these settings. Consistent with previous studies conducted in lowland and highland populations, our results support an inverse dose-response relationship between total PA volume and the odds of CVD (19, 20). A recent study by Liu et al. in middle-aged and older Chinese adults also reported that higher PA levels were associated with reduced odds of heart disease, reinforcing the protective role of PA in the Chinese population (21). However, the novelty of our study lies in its intensity-specific analysis in a high-altitude context. Nevertheless, few studies have distinguished between MPA and VPA using the most recent 2024 Compendium of Physical Activities (22). In the current study, we found that VPA was cross-sectionally associated with lower odds of prevalent CVD. This may be particularly relevant in plateau environments, where baseline cardiorespiratory stress is elevated owing to hypoxic conditions. VPA, such as carrying uphill or heavy manual labor, may enhance cardiovascular reserve, improve endothelial function, and reduce systemic inflammation more effectively than MPA in this context (23–25).

We also observed that total PA was significantly associated with WC and systolic blood pressure. These findings are consistent with the known physiological effects of PA on vascular function (26, 27). Chronic exposure to high altitudes induces hypoxia-driven erythropoiesis, increases blood viscosity, and elevates pulmonary artery pressure, which collectively increase cardiovascular vulnerability (28, 29). In such environments, the metabolic and hemodynamic benefits of regular PA may counteract altitude-related adverse adaptations more effectively than lower-intensity activities (30).

When comparing our results with those from general population studies, the magnitude of the observed association (OR for VPA vs. LPA: 0.27, 95% CI: 0.09, 0.80, *P*-trend = 0.025) appears larger than that typically reported in lowland settings. For example, the China Kadoorie Biobank study reported a hazard ratio of approximately 0.70–0.80 for high vs. low PA levels (31). This discrepancy could be attributed to several factors. First, the contrast between VPA and LPA in our high-altitude agricultural setting may be more extreme than in urban lowland populations, where the majority of individuals engage in light-to-moderate recreational activities (32). Second, chronic hypoxia may sensitize the cardiovascular system to the observed inverse association of VPA with prevalent CVD because the same absolute intensity represents a higher relative physiological challenge (33). Third, residual confounding owing to unmeasured factors could not be entirely excluded. Nevertheless, these findings indicated a possible enhanced association of VPA with CVD in high-altitude settings, which warrants further mechanistic research (34).

We found a significant interaction between WC and the intensity of PA regarding the odds of CVD. These results indicated that the inverse association between higher PA intensity and lower CVD prevalence was only evident in individuals with normal WC, while no clear pattern was observed in those with central obesity. Such heterogeneity across WC strata highlighted that the statistical relationship between PA intensity and CVD prevalence was not uniform and was strongly modified by abdominal obesity status. The observed pattern could be partially explained by cardiometabolic differences: individuals with normal WC may exhibit more distinct variation in PA intensity levels, which aligns with the stronger trend observed in this subgroup (35). However, the cross-sectional design of the study means these findings were vulnerable to reverse causation and health selection bias, which may contribute to the observed subgroup differences (36). Adults with underlying cardiometabolic impairment and enlarged WC may voluntarily limit strenuous physical activity due to physical discomfort or clinical advice, while those with healthier abdominal metabolic profiles were more likely to maintain regular VPA participation (37). This finding emphasized the need to account for abdominal obesity status when analyzing PA intensity and CVD prevalence, particularly in high-altitude settings where chronic hypoxia may further exacerbate WC-related cardiometabolic abnormalities.

This study had some limitations. First, the cross-sectional design is the most critical limitation, which precludes causal inference. Prevalent CVD was assessed at baseline, and reverse causation is highly plausible. Participants with existing CVD may have reduced PA (38), especially VPA, due to clinical symptoms or medical advice to avoid strenuous exertion, rather than low physical activity leading to CVD. This bias may have inflated the observed protective association of VPA. Therefore, the reported odds ratios reflect cross-sectional associations rather than the causal effects of VPA. Second, another notable limitation is the lack of objective PA measurement via accelerometry. Given that the present study focused specifically on PA intensity stratification, reliance solely on self-reported PA may introduce measurement error and limit the accuracy of intensity classification, which is an important methodological shortcoming worthy of explicit acknowledgment. Nonetheless, our study employed rigorous quality control procedures, which helped minimize potential bias related to self-reporting. Third, the outcome definition relied on self-reported physician diagnoses and lacked independent adjudication or imaging confirmation in all cases. This may have led to outcome misclassification, particularly for conditions such as pulmonary hypertension or high-altitude heart disease, which are common in plateau areas but are often underdiagnosed (29). Fourth, despite comprehensive adjustment for multiple covariates, residual confounding by unmeasured factors, such as genetic predisposition, cannot be completely ruled out (39, 40).

Despite these limitations, this study has several strengths. To our knowledge, this is one of the first large-scale studies to examine intensity-specific PA in relation to the odds of CVD in a high-altitude Chinese population using the most recent MET compendium (22). Comprehensive adjustments for dietary, lifestyle, and clinical covariates, including energy intake and intake of specific food groups, strengthened the validity of our findings. Furthermore, the inclusion of a well-characterized multiethnic cohort from a representative high-altitude setting adds to the external validity of our results (32).

In summary, our cross-sectional findings demonstrated an inverse association between VPA and prevalent CVD. However, these results cannot establish causality due to possible reverse causation. Prospective cohort studies with incident outcomes and objective PA measurement are needed to confirm whether VPA causally reduces CVD risk in high-altitude populations.

## Data Availability

The data analyzed in this study are subject to the following licenses/restrictions: We utilized data from the CMEC study. The baseline survey was conducted in 2018 and 2019. The details of the study design, sampling method, and characteristics are described elsewhere. Requests to access these datasets should be directed to wangyanjiao827@163.com.
