# Supplementary material for: Physical activity intensity and cardiovascular disease in Southwest China: a study on data from the China Multi-Ethnic Cohort study
Source: Front Cardiovasc Med. 2026 Jun 30;13:1834010. doi: 10.3389/fcvm.2026.1834010 (PMC13364560; doi:10.3389/fcvm.2026.1834010)
Supplement: Supplementary file 1 [file Datasheet1.pdf]

Supplementary Table S1. PA MET assignment table.

Type of PA	Way of PA	MET
Occupation PA		
Non-agricultural workers	Sedentary work	1.8
	Standing work	3.8
	General physical work (not too sweaty at room temperature such as plumbers, electricians, carpenters, masons, etc.)	4.5
	Heavy physical labor (easy to sweat at room temperature, such as loading, mining, steel making, etc.)	6.5
Agricultural workers	Manual work in the farming season	6.3
	Semi-mechanized work in the farming season	3.4
	Fully mechanized work in the farming season	2.4
Transportation PA	Walking	4.0
	Motorbike/battery powered vehicle	3.5
	Bicycle	6.8
	Private or public transportation (such as bus, car, underground, and ferry)	1.7
Housework PA Leisure PA	Housework activity(including childcare)	2.8
	Tai-Chi/qigong/leisure walking	3.3
	Brisk walking/health exercises/Yangge/square dance	4.2
	Running/gymnastics	7.4
	Swimming	7.2
	Ball games(basketball, table tennis,badminton, etc.)	5.5
	Apparatus sports	6.0
	Other exercise (e.g. mountain walking)	5.9
